# Treating wheat seeds with neonicotinoid insecticides does not harm the rhizosphere microbial community

**DOI:** 10.1371/journal.pone.0205200

**Published:** 2018-12-03

**Authors:** Yaofa Li, Jingjie An, Zhihong Dang, Haiying Lv, Wenliang Pan, Zhanlin Gao

**Affiliations:** 1 Plant Protection Institute, Hebei Academy of Agricultural and Forestry Sciences, Baoding, P. R. China; 2 IPM Center of Hebei Province, Baoding, P. R. China; 3 Key Laboratory of Integrated Pest Management on Crops in Northern Region of North China, Ministry of Agriculture and Rural Affairs, Baoding, P. R. China; Chinese Academy of Agricultural Sciences Institute of Plant Protection, CHINA

## Abstract

Wheat aphids damage wheat plants directly by feeding on them and indirectly by transmitting plant pathogenic viruses, both of which result in low yield and plant death. Due to their high root absorption and systemic characteristics, neonicotinoid insecticidal seed treatments are increasingly applied to control wheat aphids throughout the growing season in China. Ecological concerns are raised in some research, because neonicotinoids can persist and accumulate in soils. They are prone to leach into waterways, and are found in crop nectars and pollens, where they may be harmful to pollinators. Less information is available about the effect of neonicotinoid seed treatments on soil microorganisms. Here, we posed the hypothesis that neonicotinoids are not harmful to soil microbial communities. We tested our hypothesis by evaluating the effects of two neonicotinoids, imidacloprid and clothianidin, on soil microbiomes using high-throughput sequencing during three points in the wheat growth season. Except for the imidacloprid-treated soil in the seedling stage, the community richness and diversity were not affected according to Chao1, ACE and the Shannon indices, and species distribution histogram at the phylum level. However, Beta diversity indices showed that the species richness of the bacterial and fungal community was suppressed by neonicotinoids in seedling stage (high neonicotinoids concentrations), whereas by the reviving period, the changes reverted into stimulation of the soil microorganisms (low neonicotinoids concentrations). Overall, the general microbiome recovered at the end of the wheat planting season. Generally, wheat seed dressing with neonicotinoid insecticides control aphids during the entire growth period, and have no lasting adverse effects on the soil microbiome. This study provides an understanding of the influence of neonicotinoids on crop land ecology at the level of soil microbe communities.

## Introduction

Wheat is the third-largest food crop in China, particularly in the northern area, covering 24.3 million ha [1,2]. Wheat aphids, *Sitobion avenae* (Fabricius), *Rhopalosiphum padi* (Linnaeus), *Schizaphis graminum* (Rondani) and *Acyrthosiphon dirhodum* (Walker) reduce crop production annually [3]. The most dominant species is *S*. *avenae*, a migratory aphid, which attacks wheat from the Yangzi to the Yellow River region [4,5]. Wheat aphids directly damage crops by sap ingestion, desiccation of leaves and ears, and reduced germination potential. They inflect indirect crop damage by transmitting plant pathogenic viruses, particularly the wheat yellow dwarf virus (WYDV), which further reduces yield [5,6].

Foliar aphidicide sprays, such as organophosphates, pyrethroids and neonicotinoids have been the main aphid control technology for years because more modern technologies are lacking [7–9]. Another approach to insect pest management, film coating and pelleting with systemic insecticides, called ‘seed dressings’ is used to control foliar sucking pests [10–12]. Compared with the foliar spray insecticides, seed dressings have many advantages such as easy operation, lower labor costs, and lower environmental risks [10]. Because of the high root absorption and systemic characteristics, neonicotinoid insecticides were widely applied to seeds as root treatments, particularly seed dressing for maize [13,14], wheat [15,16], soybean [17], and cotton [18–21]. Of the available neonicotinoids, imidacloprid seed dressing efficiently controls wheat aphids throughout the cropping season and increases wheat production [15,22]. Thiamethoxam and clothianidin seed dressings, but not nitenpyram, acetamiprid or dinotefuran, also provide efficacious control [15,16, 23,24].

Neonicotinoid seed treatments are increasingly applied to control wheat aphids during cropping seasons in China, and they are used in IPM programs for wheat aphid management. These treatments exert no adverse effects on ladybirds, hoverflies or parasitoids, and they lead to increased spider–aphid ratios, which promotes spider-driven biocontrol services [23]. A concern is that neonicotinoids persist and accumulate in soils and have the capacity to leach into waterways[25–26]. As systemics, they transfer from soils into nectar and pollen of treated crops, where they can threaten beneficial insect species, such as pollinators and parasitoids [25–27].

The microbial community of the rhizosphere is one of the primary factors that determine plant health [28]. Microbes act in biological, chemical and physical processes to maintain healthy and stable microenvironments for plants [29]. Information on how neonicotinoids influence microbial communities is necessary to understand possible problems these insecticides may exert on cropping systems. Because such problems have not yet arisen, we posed the hypothesis that neonicotinoids are not harmful to soil microbial communities. Here, we report on outcomes of experiments designed to test our hypothesis.

## Materials and methods

### Insecticides and wheat variety

The neonicotinoids, imidacloprid 70% ZF and clothianidin 60% SC, were purchased from Hebei Veyong Bio-Chemical Co., Ltd (Shijiazhuang, China). Wheat seeds (cultivar JiMai 22), were obtained from the Shandong Academy of Agriculture Sciences, China.

### Experimental design and samples collection

The trial experiment was conducted in sandy loam soils at the Agricultural Research Farm of Hebei Plant Protection Institute, Baoding (38.572°N, 115.264°E) from October 2016 to June 2017. The base fertilizer (Hubei Aotel Chemical Co., Ltd.) with 750kg/ha applied before sowing, and irrigated three times according to local cultivation habits, on 15 November 2016, 26 March 2017, 30 April 2017. In the field, wheat seeds were treated with either imidacloprid or clothianidin coatings at 240 a.i. g/100 kg seeds, respectively. This a.i. concentration correctly models routine field application rates [30]. Untreated seed provided the control group. Field trials were studied in plots, each treatment consisted of three biologically independent replicates. each 30 m^2^. This nine-month period far exceeds the established imidacloprid half-life in fields [31], but the experimental period was extended to model a typical growing season and to consider the possibility that imidacloprid by-products may influence the rhizosphere microbiome. No other pesticides were used during the trail.

The rhizosphere soil samples were obtained from the three biologically independent replicate plots at the wheat seedling stage (SS, November 3, 2016), the reviving period (RE, March 17, 2017) and the before harvest period (BH, June 7, 2017). The samples were transferred to the laboratory in ice chests and frozen at -80°C for DNA extraction.

### Soil DNA extraction and high-throughput sequencing

Total DNA was extracted from samples using a Power Soil DNA Isolation Kit (MOBIO Laboratories) according to the manufacturer's protocol. The concentration and DNA quality were measured with an Eppendorf Biophotometer Plus (Eppendorf, Germany), and the extracted DNA was stored at -20°C for downstream analysis.

For each sample, the primers 338F (5'- ACTCCTACGGGAGGCAGCA-3') and 806R (5'- GGACTACHVGGGTWTCTAAT-3') were used to amplify the V3-V4 region of the bacterial 16S rRNA gene and the primers ITS1 (5′ -CTTGGTCATTTAGAGGAAGTAA-3′) and ITS2 (5′ -GCTGCGTTCATCGATGC-3′) were used to amplify the fungal ITS1 region. The DNA was amplified using two rounds of PCR. The PCR product from the first step were purified through VAHTS DNA Clean Beads (Vazyme, Nanjing, China). The PCR product from the second step were quantified by Quant-iT- dsDNA HS Reagent and pooled together for high-throughput sequencing using an Illumina Hiseq 2500 platform (2×250 paired ends) at Biomarker Technologies Corporation, Beijing, China.

### Bioinformatics analyses

The raw sequencing data were merged using FLASH [32] and assigned to each sample according to their unique bar codes. High-quality reads were prepared for bioinformatics analysis, and all of the effective reads from each sample were clustered into operational taxonomic units (OTUs) based on a 97% sequence similarity using UCLUST [33]. The phylogenetic affiliation of each 16S rRNA gene sequence was analyzed using the RDP [34] Classifier (http://rdp.cme.msu.edu/) against the SILVA (SSU128) [35] 16S rRNA database with a confidence threshold of 80%. The ITS sequencing data were classified using Unite (Release 7.2 http://unite.ut.ee/index.php) [36]. OTU richness (ACE, Chao1), Shannon and Simpson diversity indices were calculated in Mothur (v. 1. 35. 0) [37].

All data were analyzed using the SPSS 17.0 statistical software package, and significance was assigned at P < 0.05 using one-way analysis of variance (ANOVA) with Duncan’s tests. Permutational multivariate analysis of variance (PERMANOVA) was performed to evaluate significant differences in the microbial community composition. The Bray Curtis algorithm was used to calculate hierarchical cluster trees based on subsample files. Heat maps based on the retained OTUs and boxplot of beta diversity were created using R (version 3.0.2) with the g-plots package.

## Results

### Sequencing results and microbial diversity analysis

After quality filtering, sequencing-based analysis generated 1,794,840 bacterial 16S rRNA gene sequences and 1,941,857 fungal ITS sequences from 27 rhizosphere soil samples, with an average of 66,476 ± 1,663 bacterial sequences per soil sample and 71,921 ± 289 fungal sequences per soil sample. Based on a 97% nucleotide sequence identity between the reads, 6,311 bacterial OTUs and 3,731 fungal OTUs were identified. Rarefaction curve analysis at 3% dissimilarity level for the soil samples showed that the curves started to plateau, implying that the sampling was sufficient and reasonable (S1 Fig).

The Chao1 and ACE indices of the fungal community in the SS soils were significantly different, and indices from the imidacloprid-treated soil were significantly lower relative to controls (Table 1). No significant differences were detected in bacterial communities between the soils treated with insecticides and the control soils at any of the three time points.

**Table 1:** The characteristics of rhizosphere soil microorganism in different wheat growth periods after seed dressing.

Treatment	The soil microbial community characteristics during the indicated wheat growth period																	
	Seedling stage						Reviving period						Before harvest period					
	Bacterial community			Fungal community			Bacterial community			Fungal community			Bacterial community			Fungal community		
	ACE	Chao 1	Shannon	ACE	Chao 1	Shannon	ACE	Chao 1	Shannon	ACE	Chao 1	Shannon	ACE	Chao 1	Shannon	ACE	Chao 1	Shannon
CK	1,578a	1,580a	5.86a	505a	515a	4.29a	4,212a	4,145a	6.94a	705a	708a	4.79a	1,610a	1,613a	5.98ab	460a	465a	3.70a
IM	1,608a	1,611a	5.76a	424b	425b	3.86a	4,196a	4,166a	6.91a	709a	714a	4.86a	1,606a	1,613a	5.99a	459a	467a	3.89a
CL	1,608a	1,613a	5.94a	468ab	471ab	3.90a	4,198a	4,190a	6.90a	701a	710a	4.45a	1,600a	1,605a	5.94b	445a	452a	3.83a

The means of the Chao 1, ACE and Shannon indices of rhizosphere soil challenged with IM (imidacloprid) and CL (clothianidin) at 97% similarity. Different letters in each column indicate statistically significant differences based on Duncan’s test (*p* < 0.05).

For the fungal community, no significant differences were detected between experimental and control soils during the RE and BH.

### Community structure and PCA analysis

**Fig 1:**
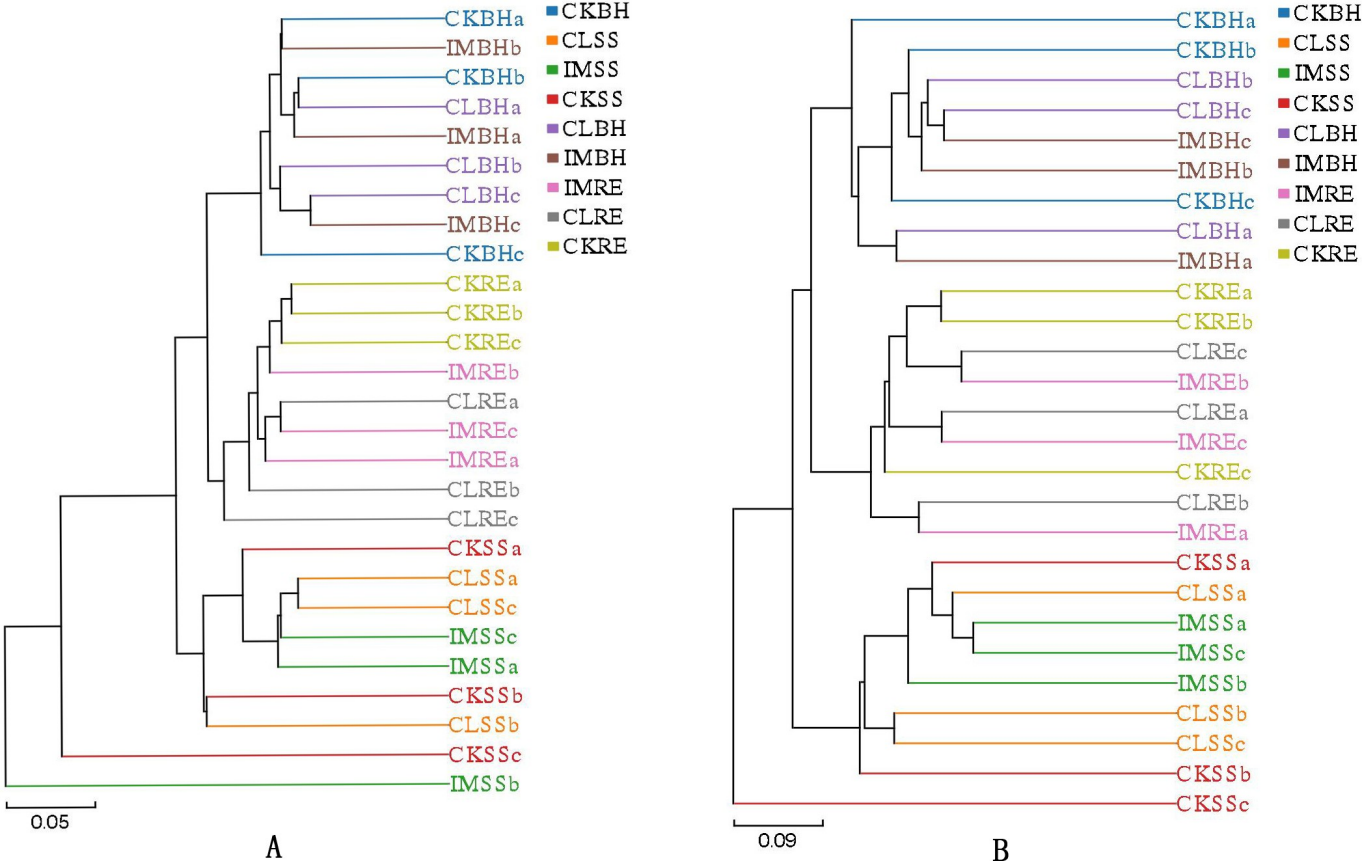
Hierarchical cluster tree of microbial communities in treated soil samples. Hierarchical cluster tree constructed based on a distance matrix calculated using the Bray Curtis algorithm for the soil samples collected from the 3 treatment conditions: CK, untreated plants (control); IM, plants challenged with imidacloprid (240 a.i. g/100 kg seeds); and CL, plants challenged with clothianidin (240 a.i. g/100 kg seeds) for 3 different growth stages of wheat plants: SS, seedling stage; RE, reviving period; BH, before harvest period. (A) bacteria; (B) fungi. The different letters (a, b, c) after the letters for all treatments indicate the three replicates.

Hierarchical clustering analysis showed that the bacterial and fungal communities collected from the soils of the same wheat growth period clustered together (Fig 1). The results were consistent with performance of the PCA analysis (S2 Fig).

### Effect of neonicotinoid insecticides on the soil microbial community composition

**Fig 2:**
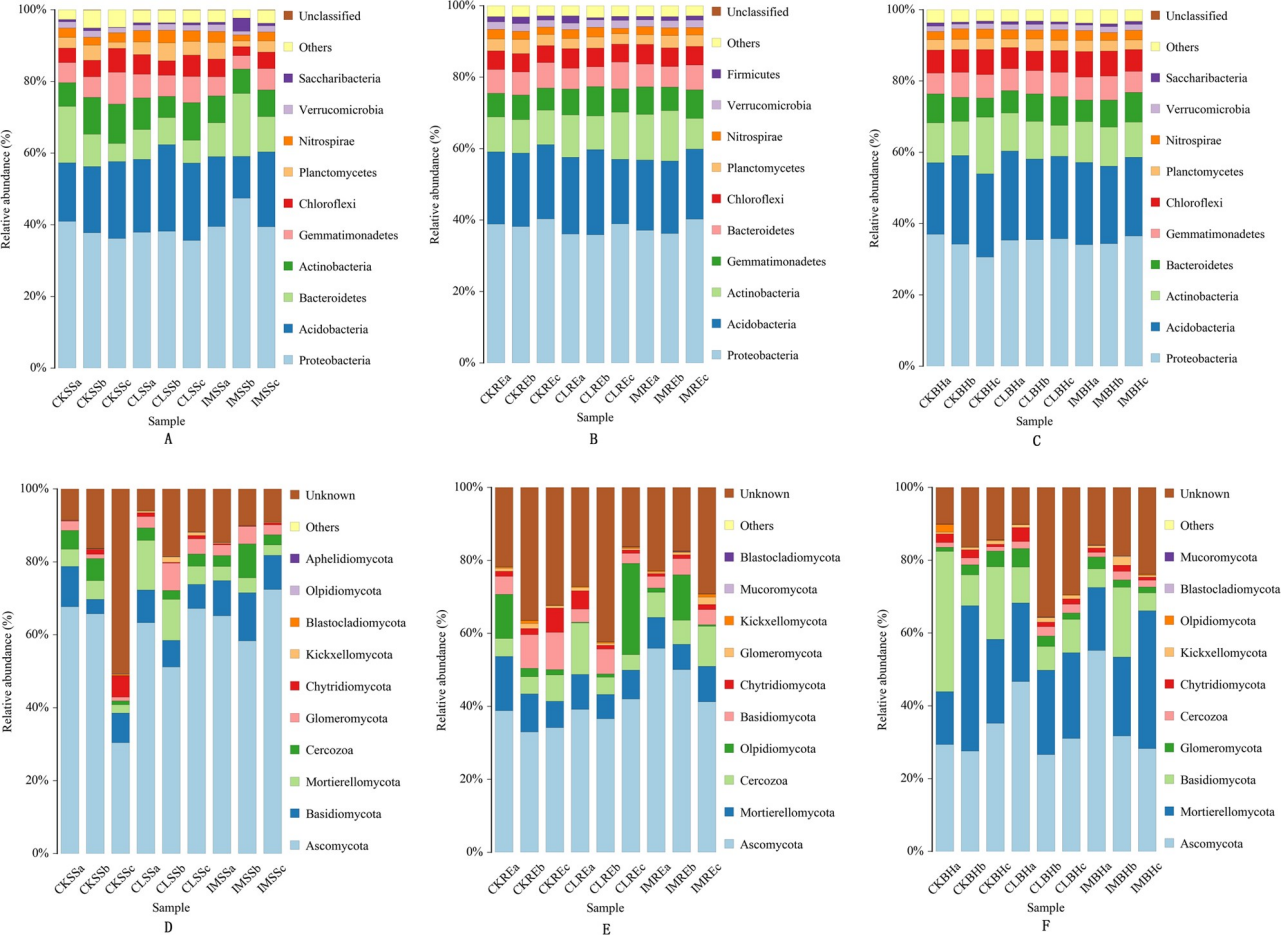
The relative abundance of dominant bacterial and fungal phyla in treated soil samples. A: bacterial phyla of soils in SS; B: bacterial phyla of soils in RE; C: bacterial phyla of soils in BH; D: fungal phyla of soils in SS; E: fungal phyla of soils in RE; F: fungal phyla of soils in BH. The dominant bacterial phyla (A, B and C) and fungal phyla (D, E and F) were from the soil samples collected from the 3 treatment conditions: CK, untreated plants; IM, plants challenged with imidacloprid (240 a.i. g/100 kg seeds); and CL, plants challenged with clothianidin (240 aig/100kg seeds) for 3 different growth stages of wheat plants: SS, RE, BH. The relative abundance was based on the proportional frequencies of those DNA sequences that could be classified at the phylum level. The different letters (a, b, c) after the letters of all treatments indicate the three replications.

Bacterial and fungal phyla were identified (Fig 2). For bacterial phyla, the relative abundances of *Proteobacteria*, *Acidobacteria*, *Bacteroidetes*, *Actinobacteria* and *Gemmatimonadetes* exceeded 80% in all samples of different wheat growth stages, with *Proteobacteria* was the most abundant phylum. We could find no evidence of the insecticide treatments on the abundances of the bacterial phyla. The relative abundances of the unclassified and other bacteria were less than 5%, showing that most of the bacterial phyla were measured in the analysis. For fungal phyla, the relative abundances of *Ascomycota*, *Basidiomycota* and *Mortierellomycota* were 50, 75 and 80%, respectively. *Ascomycota* was the most abundant phylum. Compared with the classified fungal phyla, the unclassified fungi occupied a large proportion of the samples, specifically in RP soils. Again, we recorded no discernible influence of neonicotinoids insecticides on the abundances of the fungal phyla.

**Fig 3:**
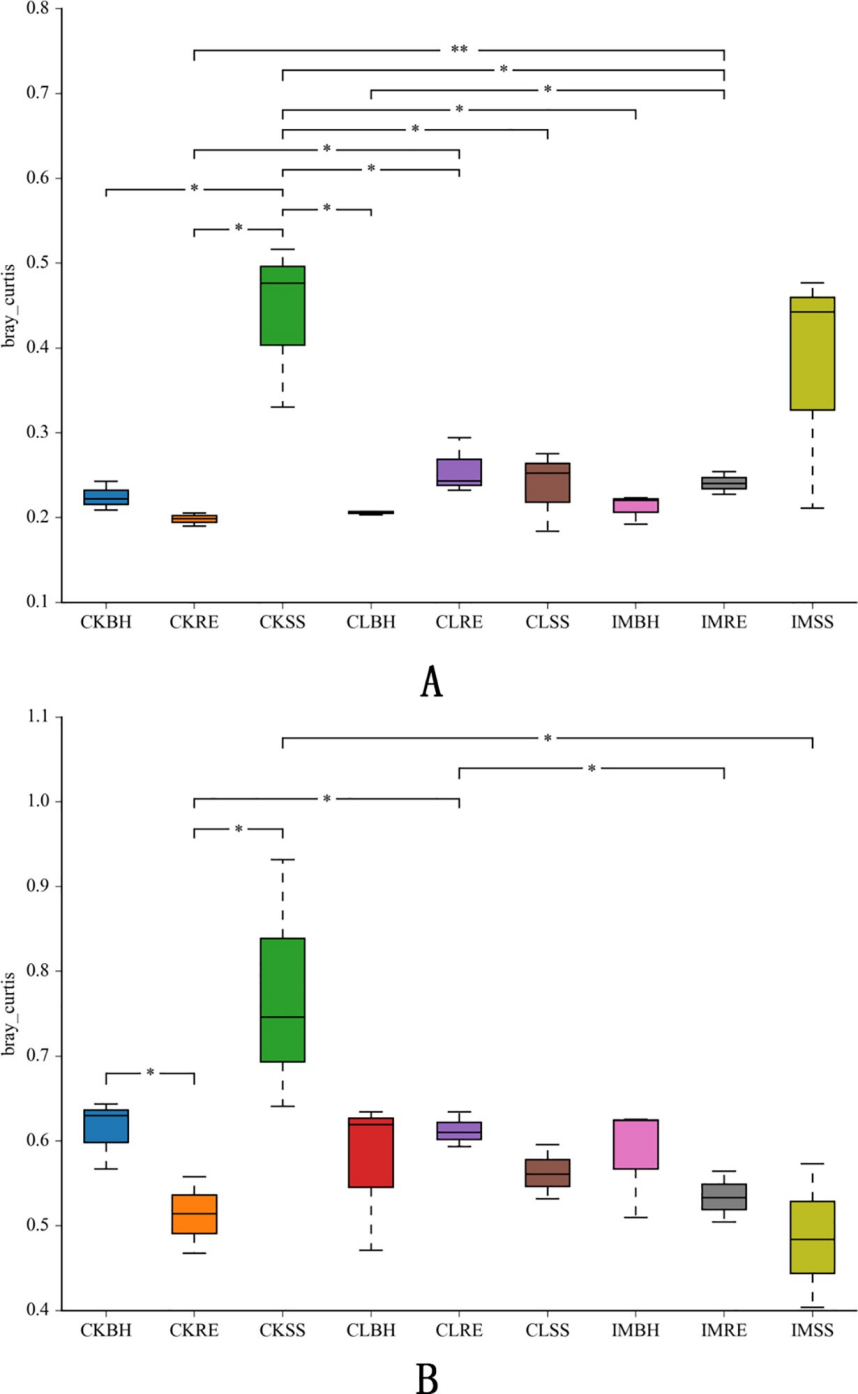
**Dis matrix box plot of the dominant bacterial (A) and fungal (B) communities.** The dis matrix box plot based on the Bray Curtis method for the soil samples collected from the 3 treatment conditions: CK, untreated plants (control); IM, plants challenged with imidacloprid (240 aig/100kg seeds); and CL, plants challenged with clothianidin (240 a.i. g/100 kg seeds), for 3 different growth stages of wheat plants: SS, RE and BH.

Differences of dominant bacterial and fungal communities in treated soils were compared by the Bray Curtis method. For the bacterial community, differences were observed between the clothianidin treatment and untreated plants in the SS soils. These differences between the pesticide treatment and untreated plants remain in the RE soils. However, we did not observe any differences in the soil bacterial communities in the BH soils. The results for the fungal communities in sample soils were almost identical to those for bacteria (Fig 3).

### Effect of neonicotinoid insecticides on BCAs

**Fig 4:**
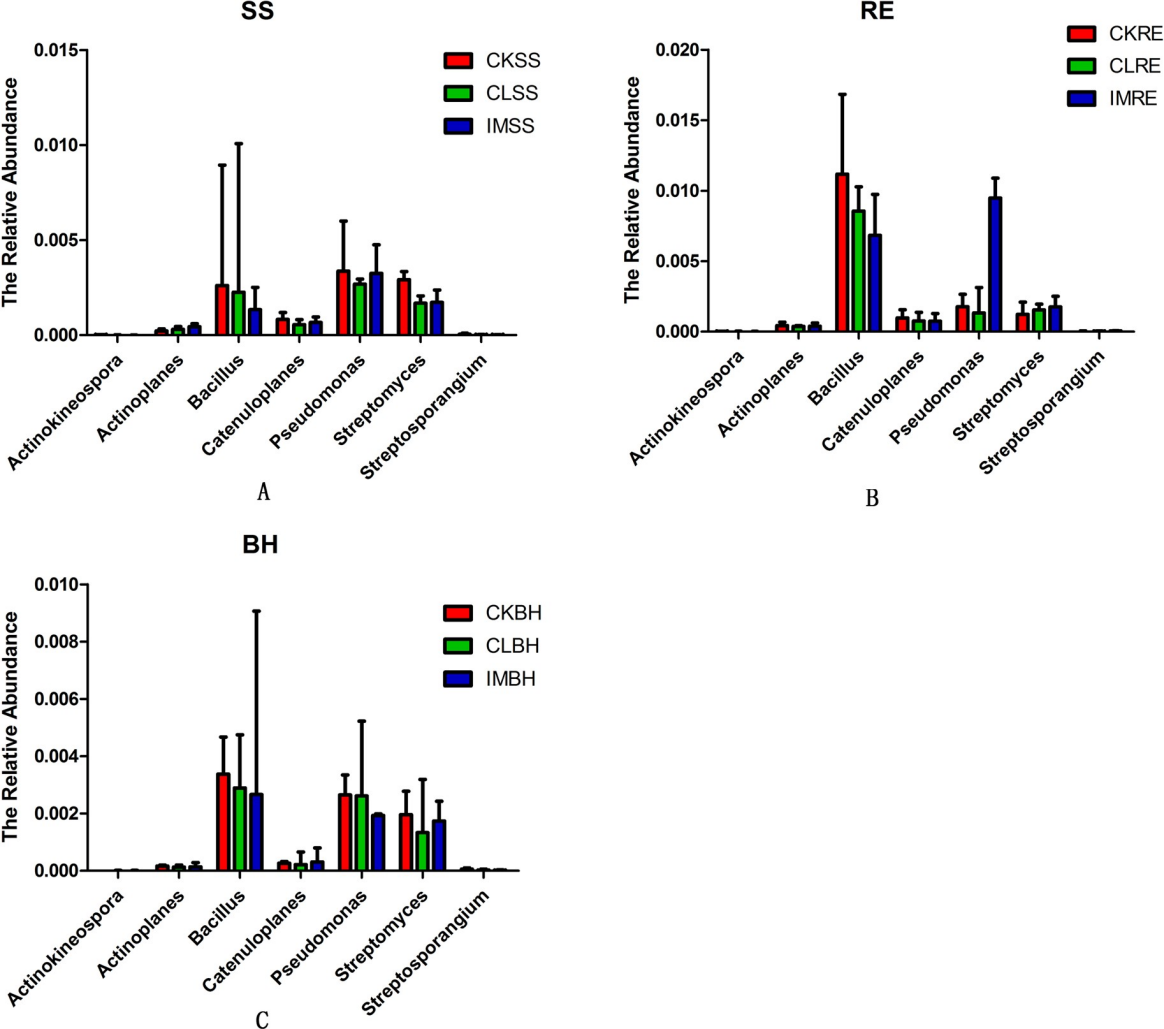
The effect of neonicotinoids on the biocontrol bacteria in soil samples. The relative abundance of the primary bacterial genera for the soil samples collected from the three treatments groups: CK, untreated plants (control); IM, plants challenged with imidacloprid (240 a.i. g/100 kg seeds); and CL, plants challenged with clothianidin (240 a.i. g/100 kg seeds) for 3 different growth stages of wheat plants: SS (A), RE (B) and BH (C).

Some biocontrol agents (BCAs) that protect plants from soil-borne pathogens and improve plant growth were chosen as representatives at the genus level (Fig 4). The relative abundance of BCAs was not influenced by the neonicotinoid insecticides in the treated soils throughout the wheat planting period.

## Discussion

With the increased use of neonicotinoids for agricultural pest control worldwide, concerns about the influence of these insecticides on agroecosystems are arising. Some issues have been investigated, particularly the direct and indirect neonicotinoid-caused mortality of non-target organisms, persistence and accumulation of the insecticides in soils, and effects on ecosystem services. The general picture indicates the neonicotionoids do not influence these issues [25, 38–41]. although impacts may occur above ground [25–27]. The data reported in this paper support our hypothesis that neonicotinoids are not harmful to soil microbial communities. Several points are germane. One, our hierarchical cluster analysis shows the insecticides did not influence the bacterial or fungal microbiomes over the growing season. Two, the relative abundances of dominant bacterial and fungal phyla were not significantly influenced by the insecticide treatments. Three, the matrix box plots similarly indicate the absence of substantial insecticidal influence. Four, the neonicotinoids did not influence populations of microbial biocontrol services. Taken together with the supplementary data, these points support our view that neonicotinoids do not influence soil microbiome populations.

The rhizosphere microbiome, a key factor in plant health and microenvironment stability, varies with respect to diversity and community composition in response to changes in the soil environment [28,29,42–44]. Some soil microbes serve as BCAs. These include several genera, Bacillus, Actinobacteria, Streptomyces, Actinospica, Catenulispora and Pseudomonas, that protect plants from soil-borne pathogens by producing antibiotics to minimize bacterial growth [45]. Given the neonicotinoids are used in soils, most organisms inhabiting arable environments will undoubtedly be exposed to, and possibly influenced by, them.

The fungal, but not bacterial, community was reduced by imidacloprid in the seedling stage. Other indices of community richness and diversity were not influenced based on Chao1, ACE and the Shannon indices, nor were the species distribution histograms altered at the phylum level. Nonetheless, there were differences among samples, seen in the box plot of beta diversity based on the retained OTUs. The bacterial and fungal community make up and richness changed during the SS and RPs, although they were reversed at the end of the wheat planting season (Fig 3). These changes differed among the growing periods. In the plant SS, the community richness and diversity of neonicotinoid treated soils were lower compared to controls, which recovered the RP stage. We infer that high neonicotinoid concentrations may suppress growth of the soil microbiome, while lower, functional doses used in agroecosystems, do not. Extending our view, we speculate the low doses used in seed dressing treatments can lead to increased microbial populations, as seen with organochlorine pesticides [46].

Soil microbiota react differently to various agricultural chemical classes and to varying concentrations of the same insecticide [46,47]. This may be due to the ability of some microbial species, but not others, to metabolize particular pesticide as a source of energy and nutrients [46]. More to the point, microbial groups may be able to metabolize smaller, but not larger, pesticide dosages, which may be toxic to them [46]. We infer that this substantial decline in soil pesticide concentrations influences the abilities of microbial communities to recover from insecticide-induced loses.

## Supporting information

S1 Fig Rarefaction curves for the soil samples. A: bacteria; B: fungi. Rarefaction analysis at 3% dissimilarity levels for soil samples obtained from the three treatments: CK, untreated plants (control); IM, plants challenged with imidacloprid (240 a.i. g/100 kg seeds); and CL, plants challenged with clothianidin (240 a.i. g/100 kg seeds) for 3 different growth stages of wheat plants: SS, RE and BH. The vertical axis shows the average number of OUTs that would be expected to be found after sampling the number of sequences shown on the horizontal axis.
(TIF)

S2 Fig The performance of soil samples by the principal components analysis. A: bacteria; B: fungi. The soil samples collected from the three treatments: CK, untreated plants (control); IM, plants challenged with imidacloprid (240 a.i. g/100 kg seeds); and CL, plants challenged with clothianidin (240 aig/100kg seeds) for 3 different growth stages of wheat plants: SS, RE and BH.
(TIF)
