# Supplementary figures and images for: Treating wheat seeds with neonicotinoid insecticides does not harm the rhizosphere microbial community
Source: PLoS One. 2018 Dec 3;13(12):e0205200. doi: 10.1371/journal.pone.0205200 (PMC6277090; doi:10.1371/journal.pone.0205200)

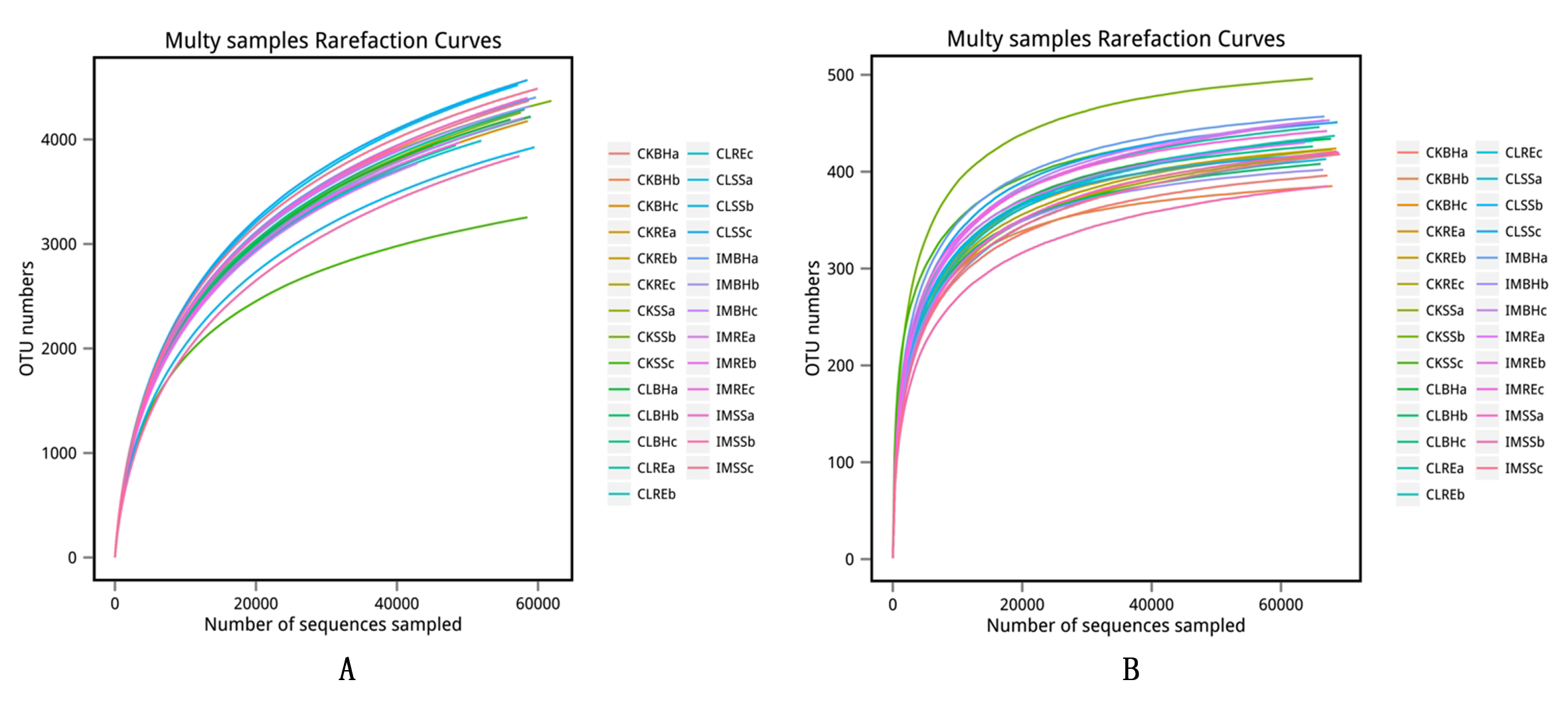

Supplement: S1 Fig — A: bacteria; B: fungi. Rarefaction analysis at 3% dissimilarity levels for soil samples obtained from the three treatments: CK, untreated plants (control); IM, plants challenged with imidacloprid (240 a.i. g/100 kg seeds); and CL, plants challenged with clothianidin (240 a.i. g/100 kg seeds) for 3 different growth stages of wheat plants: SS, RE and BH. The vertical axis shows the average number of OUTs that would be expected to be found after sampling the number of sequences shown on the horizontal axis. (TIF) [file pone.0205200.s001.tif]

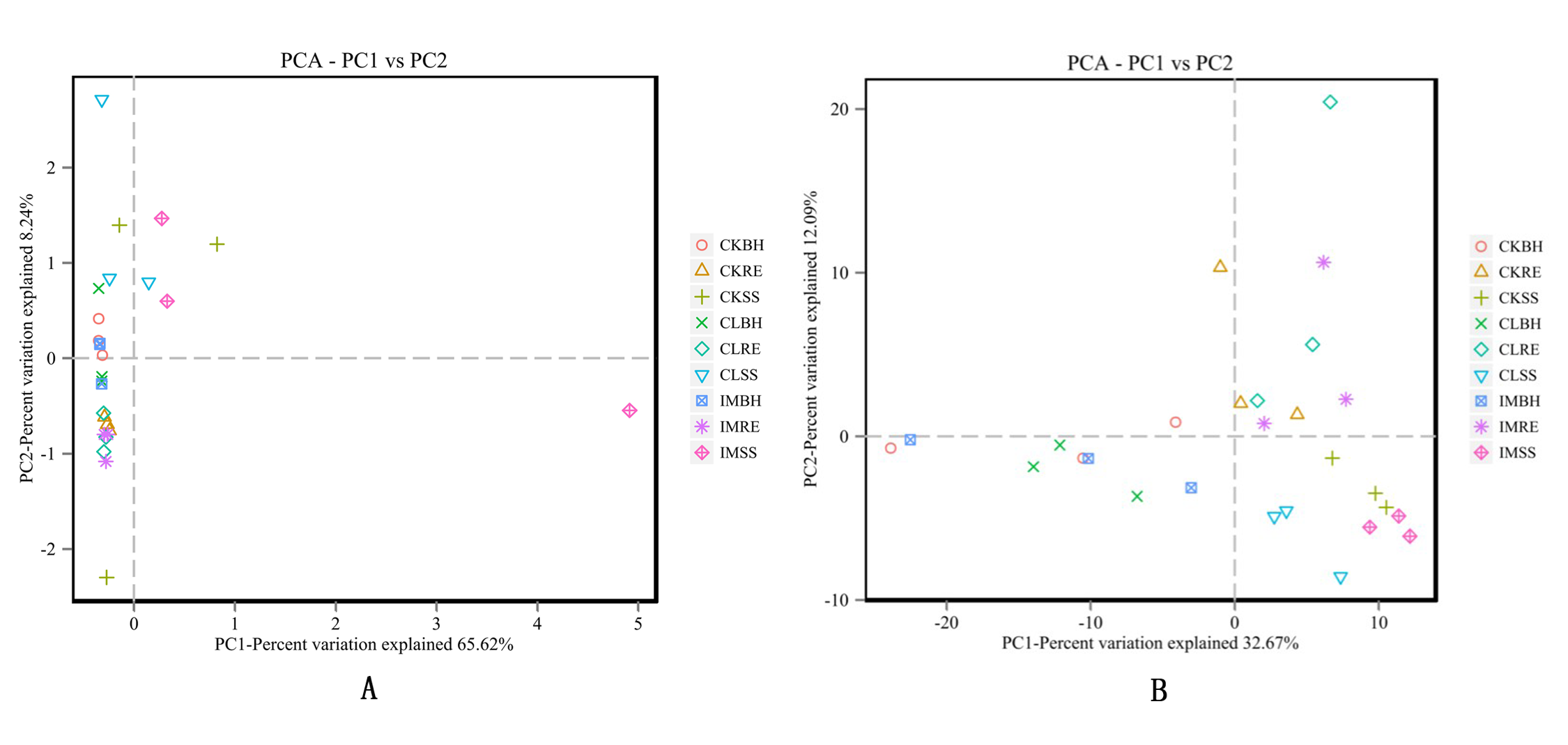

Supplement: S2 Fig — A: bacteria; B: fungi. The soil samples collected from the three treatments: CK, untreated plants (control); IM, plants challenged with imidacloprid (240 a.i. g/100 kg seeds); and CL, plants challenged with clothianidin (240 aig/100kg seeds) for 3 different growth stages of wheat plants: SS, RE and BH. (TIF) [file pone.0205200.s002.tif]
